# Debating stereotypes: Online reactions to the vice-presidential debate of 2020

**DOI:** 10.1371/journal.pone.0280828

**Published:** 2023-01-25

**Authors:** Diane H. Felmlee, Chris Julien, Sara C. Francisco

**Affiliations:** 1 Department of Sociology and Criminology, Pennsylvania State University, University Park, Pennsylvania, United States of America; 2 Population Research Institute, Pennsylvania State University, University Park, Pennsylvania, United States of America; 3 Department of Sociology, Grinnell College, Grinnell, Iowa, United States of America; HEC Montreal, CANADA

## Abstract

The 2020 Vice-Presidential debate afforded the opportunity to examine online reactions toward a woman of color, Kamala Harris, and a white man, Mike Pence, as they vied for the same position. We collected tweets from the Twitter API related to Harris and Pence, mainly using neutral hashtags. We examined keywords for gender and race slurs and conducted a multivariate analysis of tweet sentiment. Gender and racial slurs surface in both Harris and Pence datasets, showcasing the insidious nature of sexist and racist stereotypes that seep into online conversations regarding a high-status job debate. As anticipated, tweets regarding Harris contained a higher proportion of racist and sexist curse words, highlighting greater levels of harassment and “intersectional,” multi-ethnic/gender attacks. Racial insults targeting Blacks or Asians were more negative than those associated with Whites. Unexpectedly, tweets related to Harris were more positive in average sentiment than those regarding Pence. Yet, there were significantly more retweets, and more negativity of retweets, relating to Harris than to Pence, underscoring the relatively widespread broadcasting of derogatory messages about Harris. Overall, we found that harassing messages toward the candidates reinforced traditional race and gender stereotypes and bolstered the status of those who posted negative content by attaining more retweets. Harassers routinely invoked well-worn, stereotypical insults in their attacks, especially when targeting a multiracial woman.

## Introduction

In one of the most public job interviews in the country, Vice-President Mike Pence and Senator Kamala Harris engaged in a televised debate in October 2020. We systematically examine the degree to which gender and race disparities appear in reactions on Twitter to the 2020 vice-presidential debate involving Pence, a white man, and Harris, a woman of Black and Asian American heritage, as they vie for the same position. Observing the contours of these networks of online discussions affords the opportunity to study race and gender stereotypes in a highly public venue. Given documentation of systematic effects of Twitter on news reporting [1], and of Facebook and Twitter on voting behavior [2–4], a careful study of social media commentary on the VP debate is warranted. Forms of online media not only reflect aspects of public opinion; they also shape it.

We collected tweets directly from the Twitter API related to Harris and Pence primarily using neutral hashtags that emerged on the night of the vice-presidential debate, and the weeks following. We then used regular expressions to search for keywords that signified gender and race slurs and applied a sentiment classifier to the data. The purpose of this study is to compare the sentiment of tweets oriented towards the two candidates and examine the degree to which race and gender characterizations influence the negative, or positive, content of the messages. We anticipate that although tweets regarding both Harris and Pence will contain negative content, those directed toward Harris, a woman of color, will be more negative in sentiment and include more racial/ethnic and gendered slurs. Conversely, we hypothesize that tweets about Pence will be more positive and contain fewer gender and racial slurs. We also illustrate the network spread of tweets for both candidates and anticipate that negative messages will often diffuse widely in the online world.

### Race and gender discrimination

Opportunities for advancement in the United States reflect persistent racial inequality, with discrimination based on race/ethnicity prevalent within a wide range of domains, including employment, housing, credit, and consumer markets [5]. Multiple audit studies, or correspondence experiments, document high rates of discrimination against minority applicants over a period of 50 years. For example, one study matched resumes for both black and white candidate pairs, which were then used to apply for 1,008 national jobs. The findings revealed that black candidates received significantly fewer employer responses than their white counterparts, and that even an elite university degree did not provide protection from discrimination [6]. According to a meta-analysis of 43 studies and 738 tests, furthermore, members of racial and ethnic minority groups encountered substantial discrimination in hiring in 18 countries; they faced approximately half the odds (49%) of obtaining a job interview as compared to majority group applicants [7].

Job discrimination applies to gender, as well. An audit study of 1,372 job offers found evidence of biases in favor of male over female applicants in Spain [8]. Another audit study reported that job applicants who were mothers, as compared to women who were not mothers, and as compared to men, tend to be discriminated against [9]. In addition, working women are much more likely than men to recount experiences of job discrimination [10], and, of direct relevance to this study, women are vastly underrepresented in leadership positions within United States politics and business [11].

The publicly televised debate between two candidates for the position of Vice-President represented a national job interview. In the first and only Vice-Presidential debate during the 2020 presidential election, the incumbent, Republican Vice-President, Mike Pence, and then-Senator Kamal Harris (Democrat-CA) appeared together on the stage in Salt Lake City at the University of Utah on Oct. 7. The moderator, Susan Page of *USA Today*, posed questions to each candidate with the intention of gaining information about their knowledge and qualifications for the position, with the entire interchange accessible on public television. We examine responses on Twitter to the Vice-Presidential Debate to ascertain candid reactions, in real time, on the part of a segment of everyday citizens who are active online. The accessibility of content allows us to investigate the prevalence of online harassment and the use of gender and racial stereotypes, as reflected in slurs, regarding the performances of the two candidates during this job interview.

### Online harassment

Most Americans use social media platforms. According to the Pew Research Center [12], approximately 22% of adults in the U.S. use Twitter, which makes it a popular social media site today, similar in following to Snapchat and WhatsApp, but less used than Facebook and Instagram. On average, Twitter users are younger, with a median age among U.S. users of 50, more likely to have a college degree than the general public, and identify more with the Democrat, rather than Republican, political party [12].

Harassment, hate speech, cyberbullying, and other forms of problematic messages are common occurrences on Twitter and other forms of social media. Close to two-thirds of Americans report experiences with online harassment personally [13], and over 150,000 bullying messages occur on Twitter daily [14]. Racist, sexist and homophobic content appears in numerous tweets [15,16], approaching half a million tweets per day that include sexist slurs [17]. These forms of demeaning online interactions can reap psychological and emotional harm [18,19] and cause victims to stifle their online activity [13].

The question we entertain is to what extent this type of harassing language manifests itself in messages regarding the vice-presidential candidates. We focus on “paradigmatic slurs,” which are expressions of a derisive attitude towards a group of people based on factors such as race and gender [20]. These slurs invoke negative stereotypes, typically applied in ways meant to derogate and insult people [20].

### Online harassment based on gender and race

In a nationally representative survey [21], approximately 41% of Americans report having personally experienced some form of online harassment, with 18% describing severe behaviors such as physical threats and sexual harassment. Blacks (25%) and Hispanics (10%) were more likely than Whites (3%) to report victimization because of their race/ethnicity. In a recent study [22], online harassment based on race increased dramatically for Black adults from 27% in 2018 to 42% in 2020, and in just one year, to 59% in 2021. Asian-Americans experienced the largest yearly growth in severe forms of hate and harassment, from 10% in 2020 to 17% in 2021.

Additionally, women are frequent victims of negative, online content, with an abusive or problematic message sent every 30 seconds [23]. Several studies examine the way digital forms of harassment target, and affect, women [15,24–26]. These troublesome messages often reinforce traditional feminine stereotypes, such as expectations of physical beauty, sexual “purity,” and a temperament that is pleasant, kind, and soft [27]. Online content also can aim to degrade women sexually and contain threats of sexual violence [26,28]. These negative messages align with the sociocultural ethos foundational to the social web, which derives from widespread cultural forms of misogyny [24]. Furthermore, many men view these digital spaces as primarily masculine, and for men [29]. As women join these venues, thus, there has been a misogynist backlash that attempts to “reclaim” these spaces for men alone, which contributes to the rise of online abuse and vitriol directed at women [24,30,31].

Women politicians are targeted frequently by violent online threats and misogyny, presumably aimed at dissuading them from public activity [32]. During the US presidential campaign of 2020 [33], for example, women Congressional candidates received many more abusive messages on Twitter than men, and women of ethnic minority backgrounds were targeted disproportionately. US and Canadian women politicians who were highly visible also were subjected to greater incivility in social media than men [34], although equivalent gender biases do not always surface in print venues [35,36]. A report on 300,000 posts regarding three female politicians found extensive gendered and sexualized abuse and disinformation across six social media platforms; then-Senator Kamala Harris, a mixed race, influential, and visible politician, was by far the most frequent target, representing 78% of such cases [28].

Multiple oppressions ensconced in gender and race systems can influence individuals simultaneously [37–39], and scholars emphasize the need to examine race, gender, and class as intersectional systems of societal oppression. The concept of “misogynoir” [40], for example, highlights the distinctive, interlocked blend of hostility experienced by Black women. Moreover, online abuse oriented towards women of color can be particularly pernicious. Problematic messages on Twitter disproportionately target Black women, and women of mixed-race backgrounds encounter abuse in multiple forms, including sexism, racism, and physical and sexual threats [23]. Thus, intersectional theories and research suggest that the joint experience of being a Black and Asian woman is apt to shape Harris’ online and offline experiences and societal reactions to her.

It is important to note that Harris’s racial identity influences both how Kamala perceives her own identity as well as how voters view her as a political candidate. Research on Black women and their political ambitions finds that Black women may question their ability to be successful in politics, but they often overcome these doubts as a result of support and encouragement from their peers and community [41]. For this group of women, political engagement tends to represent a form of resistance to societal marginalization and an approach to achieving greater equality [42]. Kamala Harris’s experience as a minority candidate is not only as a Black woman, however, but also as an Indian woman. Harris’s multiracial identity casts light on how multiracial identities are complicated and complex. Multiracial individuals, in particular, must manage how others perceive them [43,44]. Within political campaigns, multiracial candidates have the advantage of building partnerships with voters from multiple groups, but they are disadvantaged when appealing to those of the same race with strong racial identities [45]. For instance, some South Asian groups question Harris’s representation of them and her motivations for highlighting her Indian ancestry [46].

### Theories of online aggression

According to theories of online aggression, two basic, social psychological processes contribute to the proliferation of harmful, abusive messages [47]. These processes are fundamental to everyday, social interaction, and include the development of social norms and the evolution of informal status hierarchies, drawing from formative work on group dynamics [48]. When people interact, they engage regularly in the enforcement of social norms, for example, where norms refer to group-level evaluations of behavior that are backed by social sanctions [49]. Individuals who deviate from standards set by social norms are likely to be subject to negative consequences. In the case of online aggression or harassment, people routinely reinforce traditional stereotypes based on race, gender, age, and other characteristics [17]. Perpetrators direct their derogatory attacks at individuals believed to depart from common, stereotypical, normative expectations for behavior and deportment.

Informal, status hierarchies also emerge in social interaction, and competition for status represents a second social process that governs bullying and harassment, and one that is common both online [47] and offline [50,51]. When people interact, they vie regularly for recognition, attention, and esteem from others, which can generate conflict, especially in situations where rank remains ambiguous [52]. Within social media, one version of such competition involves the motivation to get posts noticed by others and to gain followers. Since negative Twitter comments tend to receive more retweets than their positive counterparts [27], posting derogatory messages can speed the attainment of these attention-seeking goals.

Note, too, that communication on social media sites often remains anonymous, or confers the perception of pseudo-anonymity, with posts typically originating without accompanying, formal identifiers and labels, other than a Twitter handle. As such, electronic communication has been welcomed as a tool to provide “voice to the voiceless.” At the same time, the ability to remain anonymous fuels the likelihood of aggressive responses, and seeing others model such actions amplifies aggression [53]. Online communication also facilitates targeting someone of elevated social standing, such as a politician, with minimal, if any adverse consequences such as censorship or physical retaliation.

### Twitter, politics, and online harassment

Forms of social media have revolutionized today’s political landscape [54], and widely facilitated the rise of populism [55]. An experiment with 61 million Facebook users found that political mobilization messages delivered during the 2010 presidential election directly shaped actual, voting behavior, as well as increased political self-expression and information-seeking [2]. Messages shared in the experiment affected not only the behavior of the individuals themselves, but the effects spread to their friends and to their friends of friends. Another study [3] documented that Twitter influenced the 2016 Presidential election, demonstrating the potential of this form of social media as a political platform. Twitter activity during the 2020, Vice Presidential debate is noteworthy not only because of its potential for abuse, thus, but also because such commentary can shape political outcomes.

Furthermore, although Twitter facilitates communication and information dissemination, it also can present a biased impression of public opinion. According to an investigation of public debate on Twitter [56], users who retweet far-right politics on Twitter are significantly more active, and with more visible platforms, than their more moderate or left-leaning counterparts. Reactions from relatively small minorities of individuals can appear more common than those of the majority, therefore, and give the inaccurate impression that such responses are widely acceptable. One reason we believe our study is important, therefore, is the potential for discriminatory and biased tweets to influence public opinion and political outcomes.

Twitter represents a unique source of data for this topic. Although it does not offer a representative sample from the broader population, Twitter serves as a public forum for information dissemination and debate, and one that influences news reporting [1]. Moreover, when topics or events are contentious, individuals may feel less pressure to self-censor than when confronted with traditional measures such as surveys [57,58]. Individuals’ interactions on Twitter are unprompted by any researcher, instead reflecting precisely what, how, and to whom the individuals communicate.

Despite gaps in representativeness, Twitter sentiment toward feminism also highly correlates with individual gender attitudes taken from the General Social Survey [59], a finding that is relevant to our study of gender stereotypes. In these ways, gathering data from Twitter presents unique advantages for the study of immediate reactions to the Vice-Presidential debate.

## Hypotheses

Using our dataset, we examine the following hypotheses:

1. Given the prevalence of online sexism and racism, and in light of intersectional theory, we expect that the presence of gender and racial slurs will be associated with lower tweet sentiment for both candidates, but that Harris will receive more gender and racial slurs.
2. We hypothesize that the mention of racial minority terms (e.g., black; Indian), will be associated with lower sentiment.
3. We anticipate that the content of messages directed at Kamala Harris will be more negative in overall, average sentiment than those aimed at Mike Pence.
4. Finally, we expect that both Pence and Harris will be subject to gender and race stereotyping in tweets, but that these types of messages will be more negative, on average, for Harris.

## Data and methods

### Data and search procedure

We used the Twitter API, via academic research developer accounts, to gather public tweets directly from Twitter and collected messages containing key terms and hashtags related to Harris and Pence, as shown in Table 1. We restricted our data collection to hashtags that were neutral in description (e.g., #vpdebate2020; #Harris; #Pence), in addition to one popular, comparable, negative hashtag (#kamalalies and #pencelies), to increase the standardization of our comparison of message content. Hashtags represent a common way on Twitter that individuals contribute to specific conversations; searching for a given hashtag within the platform allows the individual to see all recent tweets that have included the hashtag. Neutral Twitter hashtags frequently serve as indices, marking the individual’s tweet as joining a growing conversation [60]. In contrast, negative hashtags often do not serve only as indices. They also can represent an instance wherein individuals both join a conversation and express an opinion that aligns themselves with a particular “side”. Finally, observing the frequencies of the hashtags searched (see Table 1) and the descriptive statistics of the tweets in our dataset (see Table 3), we note that the hashtags we used to scrape tweets capture both highly visible, popular content as well as low-visibility, “ordinary” content [60]. Therefore, while employing hashtags to collect data does not yield the entire corpus of tweets that pertain to the debate, we can be reasonably sure that we have collected tweets that were intentionally entering into the “vice-presidential debate” conversation.

**Table 1:** Search keywords and frequencies in the dataset.

Keyword	Frequency
#Harris	20,865
#KamalaHarris	163,272
#Kamalalies	263
#Pence	24,147
#Mikepence	41,707
#Pencelies	4,171
#vpdebate	26,657
#vpdebate2020	8,309
Total	246,703

We utilized the R package, “rtweet”, to collect our data from the Twitter API [61]. Our main dataset is comprised of nearly 250,000 interactions, including tweets, retweets, replies, and mentions. We collected data from October 2020 to November 2020 to capture tweets most relevant to the vice-presidential debate. This research was approved by the Penn State University, Office for Research Protection, Institutional Review Board (STUDY00004666). Informed consent was not required for our use of publicly available, Twitter data. We also removed personal identifiers from specific illustrations of tweets, and paraphrased the content, to provide greater anonymity.

The key terms we chose as gender and race slurs derive from the top curse words that appear on Twitter targeting men and women [62]. We include four of the most common feminine curse words, or slurs (e.g., “b*tch”), and four of the most frequent masculine curse words (e.g., “d*ck”) [62]. Note that both types of slurs, “feminine” or “masculine,” can be used to attack a woman or a man. The most common words used for race in our dataset included Black (3,846) followed by White (2,085), and the most frequent words used as racial slurs included “cracker” (36) for White race, and Hindu (99) for minority races. Anti-Hindu sentiment, or “Hinduphobia,” contributed to the common, negative use of the term, Hindu, in the data, which invoked negative stereotypes such as “Cows. crapping on the streets of Hindu USA.” The most frequent feminine slur was “b*tch” and that for masculine slurs was “b*stard” (see Table 2).

**Table 2:** Race and gender terms, with frequencies in the dataset.

Term	Frequency	Reference	Reference Total
Black	3,846	Minority Race Words	
Indian	329	Minority Race Words	
Asian	177	Minority Race Words	
Mixed	62	Minority Race Words	**4,355**
N*gger	0	Minority Race Slurs	
N*gga	22	Minority Race Slurs	
Hindu	99	Minority Race Slurs	
Pariah	1	Minority Race Slurs	**122**
White	2,085	White Race Words	
Caucasian	1	White Race Words	**2,086**
Cracker	36	White Race Slurs	
Honkey	0	White Race Slurs	
Vanilla	10	White Race Slurs	
Whitey	3	White Race Slurs	**49**
B*tch	341	Feminine Slurs	
Sl*t	8	Feminine Slurs	
Wh*re	23	Feminine Slurs	
C*nt	12	Feminine Slurs	**380**
D*ck	176	Masculine Slurs	
P*ssy	37	Masculine Slurs	
F*g	9	Masculine Slurs	
B*stard	454	Masculine Slurs	**676**

**Fig 1:**
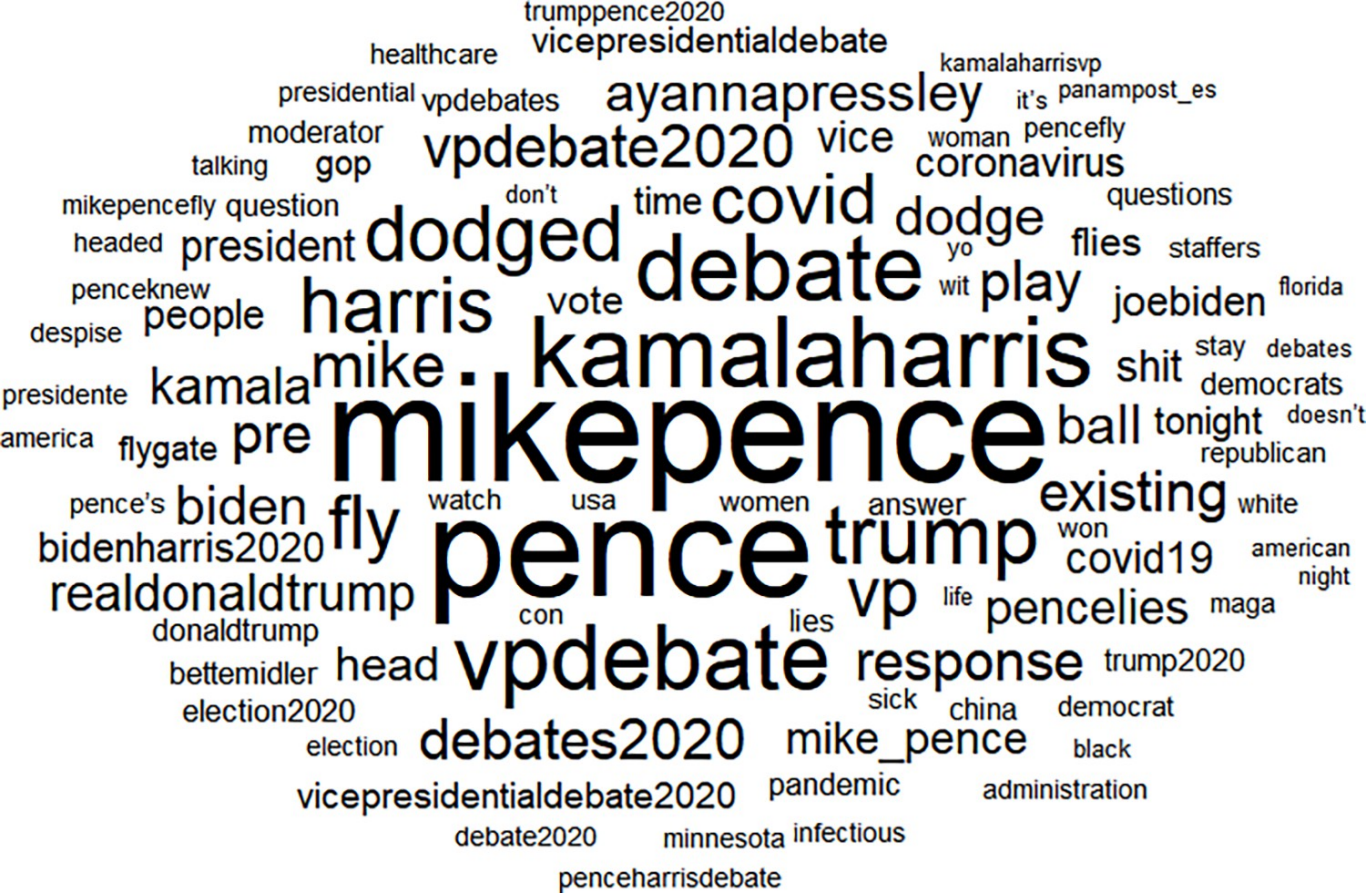
Pence top 100 words.

**Fig 2:**
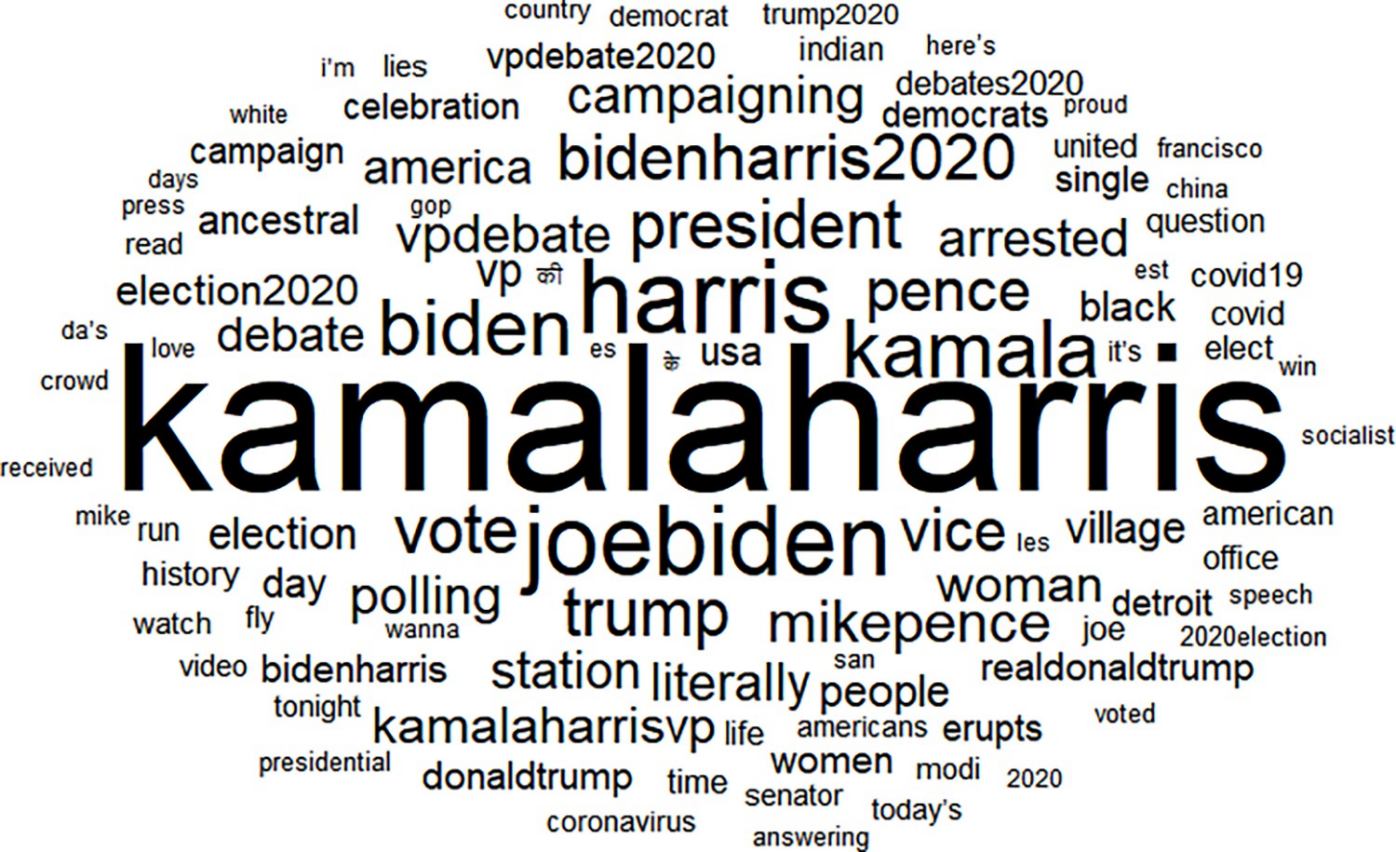
Harris top 100 words.

We present word clouds of the top 100 words found in tweets pertaining first to the incumbent, Pence (Fig 1) and next to the contender, Harris, in Fig 2 below. Note that several of the top 100 words are common between the Pence-related and Harris-related tweets, including several names (harris, pence, trump) and political keywords (vote, democrats). There are a few top words related to gender and race, including white, black, Indian, woman, mansplained, and “Imspeaking.” Several keywords arose in light of Pence’s behavior during the debate, referred to as explicit male dominance and thin-skinned, “white fragility,” and Harris’s response [63]. In addition, we find that some of the most frequently used words in the Pence dataset concerned the coronavirus (e.g. “covid”) and the fly that landed on his head during the debate (“fly”). Other recurrent words within Harris messages focused on words related to voting (e.g., “vote,” “polling”).

### Sentiment analysis

We use a supervised method for classifying the sentiment of tweets that is specifically developed to assess emotional content in online harassment on Twitter [17,64]. The classifier represents an ensemble of three popular, lexicon-based classifiers (“VADER” (https://github.com/cjhutto/vaderSentiment), “bing” [65], and “afinn”) [66]. In this approach, the word order of a tweet is ignored, and extremely common words (i.e., “stop words”) are removed. The final sentiment score is calculated as the sum of the scores of the individual words in each tweet and ranges on a scale between -4 (most negative) to 4 (most positive). When compared with sentiment scores for tweets derived from four human coders for a sample of 400 random tweets, the performance of the ensemble classifier represented an improvement over those based on VADER’s classifier alone, or over alternative combinations of the three, original, common classifiers (i.e., Vader, bing and afinn). For more details regarding the ensemble classifier see [67].

### Multivariate approach

We utilize ordinary least square (OLS) regression analysis to examine trends in the sentiment of the Twitter dataset. The primary predictors in our analyses consist of gender and race references, shown in Table 2. These variables are binary indicators that take a value of “1” if the tweet contained a gender reference or a race reference in the data (see Table 2), or “0” if the tweet did not contain such a reference. We likewise have “Pence” and “Harris” variables, which indicate if the individual’s first or last name is in the tweet. The dependent variable in our analyses is the sentiment score of a given tweet.

We also include a set of control variables in our analyses: *logged retweets*, *logged friends*, *logged followers*, and *logged favorites*. Retweets are instances where an individual shares a tweet, typically authored by another user, to their own Twitter profile. Friends are those individuals for whom the user has chosen to receive updates; activity of “friends” is shown on the user’s “timeline.” In contrast, followers are individuals who choose to receive updates about the user. Therefore, friends are those ties emanating *from* the user, while followers are those ties directed *towards* the user. Finally, favorites appear as a heart icon on tweets and, broadly, indicate acceptance of the post. We use a natural log transformation of these four variables, since their distributions are skewed, which is typical of datasets containing viral tweets.

### Robustness

In analyses not shown here, we compared results from this modeling approach to others, including ordered logit and proportional odds regression. Our findings were consistent with the OLS regression, where the same coefficients achieve significance, have similar magnitude, and comparable AIC scores. For these reasons, we proceed with the least complex, multivariate approach, the OLS regression. Findings were reported to three decimals to enable the recovery of more precise p-values, if warranted [68]. There was no evidence of multicollinearity among our independent variables, and no variable had a variance inflation factor (VIF) above 2.5. Finally, using a random sample of 6,510 accounts from our dataset, we found that less than 10% were identified as bot accounts, according to Botometer (version 4), a tool to detect social bots on Twitter [69]. Their inclusion as a control variable in a narrowed dataset did not substantively change our findings.

## Results

### Descriptive information

We begin by comparing patterns in the descriptive statistics for tweets associated with Pence and Harris (see Table 3). Findings from t-tests indicate that the majority of our variables of interest in the Pence and Harris datasets are significantly different from each other (Welch’s Two Sample). A significantly greater proportion of tweets in the Harris dataset, compared to the Pence dataset, contain common racial words and slurs for minority individuals only, as well as more masculine slurs (minority race words: Harris (2.03%), Pence (1.37%)); (minority race slurs: Harris (.05%), Pence (.04%)); (masculine slurs: Harris (.32%), Pence (.11%)). This confirms hypothesis one, that tweets regarding Harris will contain a greater number of race and gender slurs than those regarding Pence. For feminine slurs, as well as white race words and white race slurs, there is no significant difference in the proportion of tweets containing these terms. Interestingly, while the Pence dataset contains slightly fewer masculine slurs compared to feminine slurs (.11% versus .16%), the Harris dataset contains just over twice as many masculine slurs as feminine slurs (.32% versus .16%).

**Table 3:** Descriptive statistics.

	Overall	Pence	Harris
Sample Size	246,703	79,622	194,018
Retweets (sd)	1,848 (5,211)	546 (1,643) ***	2,124 (5,759) ***
Friends (sd)	2,281 (7,153)	2,204 (7,069) ***	2,312 (7,025) ***
Followers (sd)	12,922 (249,002)	13,764 (261,714)	14,010 (259,968)
Likes (sd)	2.85 (166)	2.65 (143)	2.83 (164)
Logged Retweets (sd)	3.21 (3.36)	2.12 (2.87) ***	3.34 (3.39) ***
Logged Friends (sd)	6.49 (1.71)	6.45 (1.74) **	6.48 (1.73) **
Logged Followers (sd)	6.08 (2.21)	6.03 (2.25) ***	6.1 (2.23) ***
Logged Favorites (sd)	.21 (.63)	.25 (.64) ***	.22 (.64) ***
Sentiment (sd)	.04 (1.19)	-.02 (1.16) ***	.06 (1.20) ***
Minority Race Words (%)	4,355 (1.77%)	1,089 (1.37%) ***	3,931 (2.03%) ***
Minority Race Slurs (%)	122 (.05%)	28 (.04%) *	102 (.05%) *
White Race Words (%)	2,086 (.85%)	667 (.84%)	1,630 (.84%)
White Race Slurs (%)	49 (.02%)	12 (.02%)	40 (.02%)
Feminine Slurs (%)	380 (.15%)	124 (.16%)	316 (.16%)
Masculine Slurs (%)	676 (.27%)	84 (.11%) ***	612 (.32%) ***

p-values: < .05*, < .01**, < .001***, indicating a significant difference between the Pence and Harris subsets (Welch’s Two Sample T-Test).

### Multivariate analyses

From our multivariate analysis results, shown in Table 4, we see that logged retweets are significantly, and negatively related to tweet sentiment in Model 1. Next, we find that the sentiment of tweets containing minority racial slurs, as compared to those without such slurs, is significantly more negative (Model 2), which confirms our second hypothesis. Tweets with white race words are significantly more negative than those that do not contain such words, while those with white race slurs are more positive. The control variable, logged friends, is significant and negative, logged followers is significant and positive in the model, whereas logged likes is nonsignificant. In Model 3, we see that gender slurs contribute significantly and negatively to the regression.

**Table 4:** Full dataset regression of tweet sentiment.

	Model 1	Model 2: race	Model 3: gender	Model 4: Pence and Harris	Model 5: All
**Intercept**	.165***	.172***	.175***	.261***	.268***
**Logged Retweets**	-.038***	-.039***	-.038***	-.043***	-.043***
**Logged Friends**		-.008***	-.006**	-.006**	-.005*
**Logged Followers**		.007***	.006***	.005**	.005**
**Logged Likes**		.002	.001	.002	.001
**Min_race_words**		.014			-.006
**Min_race_slurs**		-.445***			-.422***
**White_race_words**		-.060*			-.067*
**White_race_slurs**		.580**			.580**
**Fem_slurs**			-1.533***		-1.545***
**Masc_slurs**			-2.602***		-2.629***
**Pence**				-.171***	-.177***
**Harris**				-.022**	-.020**
**R** ^**2**^	.012	.012	.028	.016	.032
**Adj. R** ^**2**^	.012	.012	.028	.016	.032
**AIC**	781,427.1	781,387.3	777,466.4	780,496.9	776,426.9
**BIC**	781,458.4	781,491.5	777,549.8	780,580.2	776,572.7

p-values: < .05*, < .01**, < .001***.

Next, according to findings in Model 4, the tweets including a mention of either Pence or Harris are significantly more negative in sentiment than those without these names, although the model fit is not as good as the previous ones that include race or gender variables. Moreover, while the sentiment score of tweets with a Harris-keyword are more negative than those without such a keyword, tweets with a Pence-keyword are even more negative, while controlling for other variables, which is contrary to our third hypothesis. Finally, including all key variables in Model 5 represents an improvement over earlier models, with the lowest AIC and BIC values, and the highest adjusted R^2^. Our variables of interest remain significant in Model 5, with much of the effect on sentiment deriving from the presence of minority racial slurs, feminine slurs, and masculine slurs, which negatively relate to sentiment, and white racial slurs, which have a positive relationship to sentiment. This is consistent with patterns in prior models.

To elucidate the possible differences between the Pence and Harris corpora of tweets, we split this dataset, filtering tweets related to each candidate. This was accomplished by using regular expressions to search for instances of the candidates’ first or last names. In this way, tweets could be assigned to the Pence dataset if they contained “mike” or “pence,” to the Harris dataset if they contained “kamala” or “harris,” or both if they contained either the first or last names of both candidates. As seen in the best fitting model, Model 5 (Table 5), Pence-related tweets that contain minority racial words are significantly more positive in sentiment than those without such words, whereas those with white racial words are more negative. Tweets with masculine and feminine slurs remain significantly more negative. Likewise, for tweets regarding Harris (Model 5, Table 6), those including feminine and masculine slurs are significantly lower in sentiment. In addition, the inclusion of minority racial slurs and white racial words in Harris-related tweets results in significantly more negative sentiment, while those tweets with white racial slurs are significantly more positive. Therefore, we find that tweets in the Harris corpus drove the effects of white race slurs in the full dataset. In both split datasets, as in the full dataset, moreover, minority race slurs and white race words are linked significantly to lower sentiment scores. To test if the two hashtags “#pencelies” and “kamalalies” drove these results, in analyses not shown here we dropped these tweets from the dataset, finding that our results did not substantively change. Therefore, we include them in the analyses and models shown and discussed here.

**Table 5:** Regressions of sentiment for tweets regarding Pence.

	Model 1	Model 2: race	Model 3: gender	Model 4: Pence and Harris	Model 5: All
**Intercept**	-.009	.048**	.060***	.051**	.053**
**Logged Retweets**	-.005**	-.004**	-.005**	-.004**	-.005**
**Logged Friends**		-.014***	-.014***	-.014***	-.014***
**Logged Followers**		.006*	.005	.005	.005
**Logged Likes**		-.011	-.010	-.011	-.010
**Min_race_words**		.212***			.213***
**Min_race_slurs**		-.495*			-.411
**White_race_words**		-.090*			-.092*
**White_race_slurs**		.299			.393
**Fem_slurs**			-1.170***		-1.178***
**Masc_slurs**			-1.244***		-1.232***
**Pence**					
**Harris**				.005	.002
**R** ^**2**^	.0001	.0010	.0032	.0004	.0037
**Adj. R** ^**2**^	.0001	.0009	.0031	.0004	.0036
**AIC**	249,343	249,291	249,109	249,330	249,074
**BIC**	249,371	249,384	249,184	249,395	249,195

p-values: < .05*, < .01**, < .001***.

**Table 6:** Regressions of sentiment for tweets regarding Harris.

	Model 1	Model 2: race	Model 3: gender	Model 4: Pence and Harris	Model 5: All
**Intercept**	.231***	.214***	.214***	.268***	.275***
**Logged Retweets**	-.051***	-.051***	-.051***	-.057***	-.057***
**Logged Friends**		.001	.001	-.002	.000
**Logged Followers**		.003	.003	.004*	.004*
**Logged Likes**		.008	.006	.007	.005
**Min_race_words**		-.0012			-.017
**Min_race_slurs**		-.464***			-.485***
**White_race_words**		-.113***			-.119***
**White_race_slurs**		.623**			.625**
**Fem_slurs**			-1.664***		-1.660***
**Masc_slurs**			-2.738***		-2.760***
**Pence**				-.213***	-.220***
**Harris**					
**R** ^**2**^	.0211	.0213	.0406	.0251	.0450
**Adj. R** ^**2**^	.0211	.0213	.0406	.0251	.0450
**AIC**	617,448	616,137	613,549	616,656	612,660
**BIC**	617,478	616,239	613,630	616,728	612792

p-values: < .05*, < .01**, < .001***.

Finally, note that the negative coefficient for number of retweets (logged) is larger for Harris (-.057) than that for Pence (-.005) in Model 5 for each of the separate analyses (Tables 5 and 6), and this difference is significant (p < .001). Not only does the average tweet in the Harris dataset have close to four times as many retweets (2,124) as the average tweet in the Pence dataset (546; p < .001, Table 3), but the retweeted messages in her dataset tend to be more negative in content. These results support our final hypothesis, Hypothesis 4. In sum, the relatively high retweet rate for tweets mentioning Harris, and the elevated negativity of these retweets, suggests that derogatory messages about Harris diffused especially widely in the virtual, Twitter sphere.

### Illustrations of popular tweets

We turn now to examples of tweets from the dataset, as well as demonstrations of particularly “viral” tweets: those with some of the highest retweets counts that spread far online. These can be positive in content, but frequently, are quite negative. These examples illustrate the distance these popular tweets can travel within Twitter, dispersing well beyond the original poster, often to tens of thousands of people or more. Illustrations of tweets are paraphrased, and identifying material removed, to preserve anonymity.

#### Pence

The top two tweets within the Pence dataset concern two very different topics: the first one references the fly that landed on Pence’s hair during the debate and is comedic in tone. As paraphrased, it reads:

There is a fly on the top of Pence’s head, i.e., “flygate” [URL].

In contrast, the 2^nd^ is concerned with Pence’s relationships, and seeks to claim that his spousal relationship is not what it should be. This tweet (paraphrased) is:

This video describes VP Mike Pence and his bizarre and disturbing marriage with his wife, Second Lady "Karen" Pence [URL]

**Fig 3:**
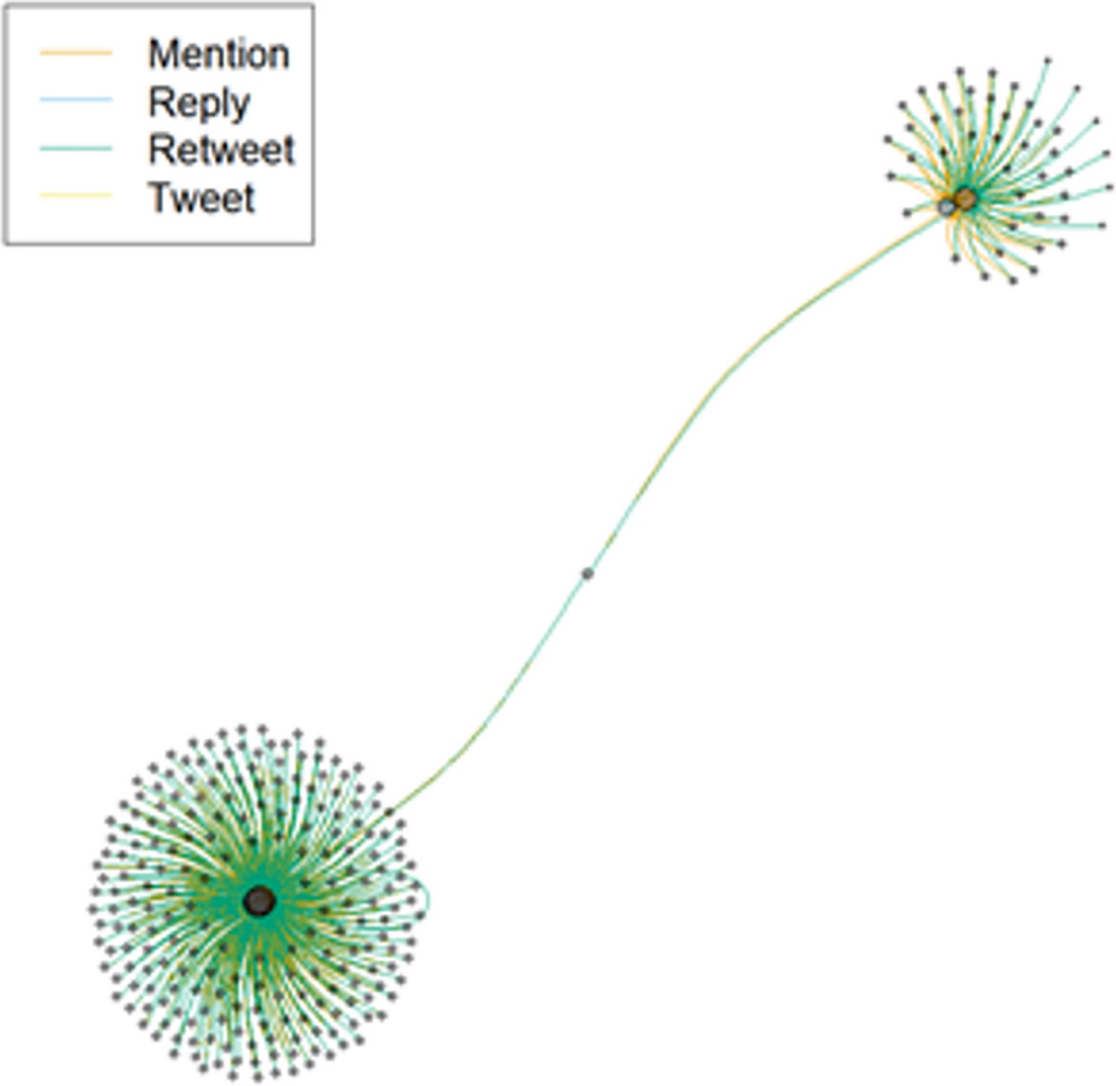
The two most retweeted tweets relating to Pence.

The network of these two tweets is shown in Fig 3. Interestingly, we can see that there is one individual who connects these two otherwise disparate hubs of activity.

#### Harris

**Fig 4:**
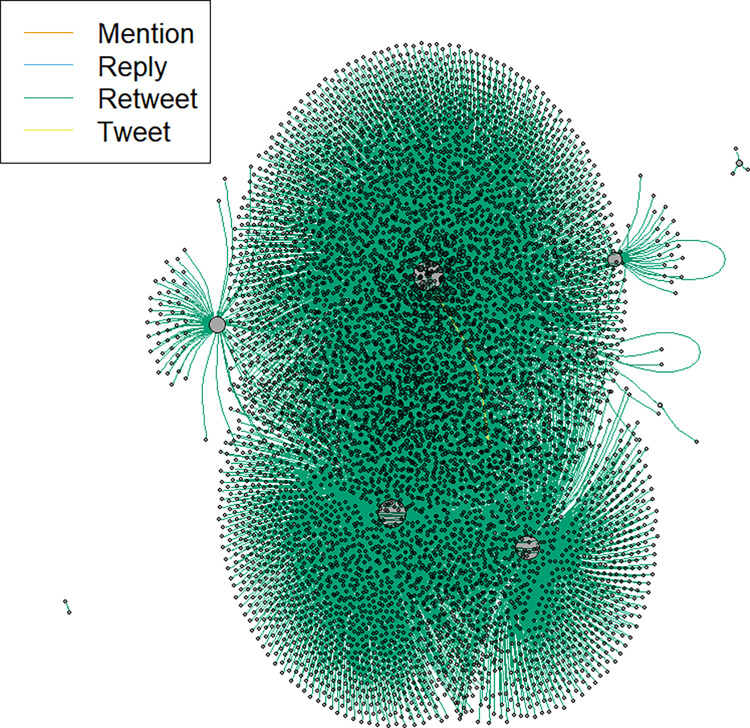
Several popular tweets related to Harris.

Several popular tweets involving Harris were opposed to her, and their networks of interactions are depicted in Fig 4. Two of these cases are as follows (paraphrased):

Wait until Election Day. This was an incredible con really. The riots were used to make Biden choose Harris for all incorrect reasons. Next the socialists will assume the presidency.

Of course, some U.S. citizens and many illegal ones will cast votes for these two China tools. Harris and “WhatsHisName”

These figures depict the extensive reach of certain instances of critical, online content.

### Illustrations of tweets and gender and race stereotypes

There were many instances of racial and gendered themes in tweets pertaining to Pence and Harris, some of which contain the slurs shown in the descriptive details of Table 2. Next, we describe illustrations of specific tweets for both the Pence and Harris datasets and note gender and race themes when they occur. Tweets are again paraphrased and identifying material removed.

#### Pence

There was a combination of positive and negative tweets regarding Pence. Some praised Pence while insulting Harris, while others only insulted Pence, such as this one (paraphrased):

You need to credit Mike Pence. Previously, white crackers were the most plain, flavorless, disgraces on Earth. Pence makes those appear as tasty ice cream.

Here, the user is focusing on what they consider to be the bland, uninteresting demeanor and personality of Pence. However, in the following example, we see another user praising Pence, while simultaneously comparing Harris to a young woman who inappropriately shares the debate stage with him (paraphrased):

In the most well-mannered effort conceivable Pence put Harris where she belongs. She appeared as a patronizing b*tch and a sulky teenage girl [URL]

Instead of critiquing his stoic demeanor, this user regards his temperament as a balm to Harris’ performance. Note, however, that the criticism of Harris here relies on gendered slurs and expectations, contrasting Pence’s performance with Harris’ inappropriate display of femininity. Finally, some tweets explicitly mention Pence’s masculinity or call it into question, as with the paraphrased example below:

@USER1 Consider the females who think VP Pence is “out of this world.” Everyone a d*minatrix? They probably feel like smacking the bej*sus out of the guy….Those who believe Pence was the winner of the debate must think the South won the Civil War

In this case, the individual suggests that women who admire Pence are those who take a dominating role during sex, suggesting that Pence is someone who takes a submissive part. Such a role is emasculating and out of line with traditional, hegemonic masculinity expectations for men.

#### Harris

There was likewise a combination of both positive and negative tweets related to Harris in our sample. Negative attacks often focused both on her gender and racial/ethnic identity, in line with intersectional scholarship. For instance, one individual tweeted the following (paraphrased):

Lies gush from the smutty, wh*rish mouth of Harris. Can’t get enough d*k to be VP.

In this example, Harris is targeted with the use of a gender slur and by the suggestion that she did not earn her position through education and work, but rather through promiscuity. In addition to implying Harris was promiscuous, individuals accused her of not being “moral enough,” and criticized her for being “ambitious,” which is most often perceived as a masculine (i.e., “unfeminine”) trait. As well as criticisms of her gender performance, she was harassed regarding her racial/ethnic identity, with some people claiming she is not Black enough, and others, that she is not Indian enough. Consider the following paraphrased tweet:

Kamala what tf will your repulsive a*s do regarding Coronavirus? What do you think, h*e? You can’t even choose a skin tone.

Here the individual slams Harris based on both gender and race, with her physical complexion the focus of the racial attack.

Another case illustrates the use of white racial language within the Harris dataset, as seen below (paraphrased):

Follow the music. Joe is falling into dementia, he is the vanilla option to get Kamala Harris, the extremist, in power.

This person uses a white racial insult to describe Biden as the boring, Caucasian candidate, invoking the stereotype of being uninteresting, bland. The user also makes an ageist attack on Biden (“dementia”) and suggests that in electing him President, Harris would ultimately be the one in power. Several other slurs in the Harris dataset target Biden.

Positive tweets about Harris, on the other hand, often congratulated Harris on her nomination as vice-president and others “called out” the adverse tweets. In one of the most popular tweets in the Harris dataset, for example, the author expresses gratitude that their daughter can look to Harris as a fitting gender/racial role model to look up to in politics, as follows (paraphrased):

Let’s pretend to be Vice President, Mom! Thankful that my young daughter views herself symbolized on a governmental podium.

## Discussion

We investigated the degree to which two candidates applying for the job of Vice-President, an incumbent white male and a mixed race, female Senator, received differing reactions on Twitter following their nationally publicized debate. We find substantial evidence of unequal treatment. For example, tweets mentioning Kamala Harris are significantly more prone to include racist and masculine slurs than those citing Mike Pence, as expected. Retweets of messages regarding Harris are much more common, in addition, and significantly more negative, than those for Pence.

Our results provide support for theories and research indicating that a woman from a minority racial and ethnic background will be treated more harshly in a job interview than a white male. Theories of intersectionality [37–39] suggest that multiple sources of oppression operate jointly to place Harris at a disadvantage, given her identities as a Black and Asian woman. In line with these expectations, the Harris corpus contains roughly three times the proportion of tweets with common curse words typically used to insult men, such as “b*stard.” Although feminine slurs appear in equal proportions in both the Pence and Harris datasets, tweets containing either feminine or masculine slurs exhibit lower sentiment scores for Harris. Minority race demographic characteristics (e.g., black, Asian), and minority race slurs, appear significantly more often in tweets concerning Harris and relate more negatively to tweet sentiment, illustrating instances of misogynoir and other forms of joint gender and race insults [40]. In messages regarding Pence, on the other hand, those with minority race words exhibit significantly more positive sentiment than those messages without such a term. Moreover, retweets of messages relating to Harris are significantly more negative than those for Pence.

A woman of color competing for this top leadership position, therefore, is subjected more frequently than a white man to insulting and abusive racist and sexist reactions. The mixed-race heritage of Harris also incurs multiple types of intersectional harassment, with attacks on her two race and ethnic heritages and on her gender. For example, we found that some messages criticized Harris’s racial identity by claiming she was not “Indian” or “Black” enough. This finding reflects the challenges that individuals with multiracial identities face, and how they must manage the perceptions of others [43,45]. Research regarding the digital harassment of public women [32], moreover, suggests that the heightened targeting of a politician such as Harris ultimately reflects perpetrators’ goals to objectify and silence active women, especially those from minority groups, and dissuade them from political life.

At the same time, tweets relevant to Harris containing a white race slur are significantly more positive than those without such a slur. Two explanations for this pattern arise. First, in comparison to tweets in this study with minority racial slurs, those with white slurs are not as negative in average sentiment. Racial insults associated with Blacks or Asians tend to contain more vitriol than those linked to being White. In addition, in several tweets using a white racial slur within the Harris corpus, the messages harass Biden, not Harris. Thus, tweets with white slurs are more positive in Harris messages than those with minority racial insults that aim to hurt her directly.

Harris also is treated less severely than Pence in the overall, emotional tenor expressed in our Twitter dataset, providing evidence counter to typical race and gender biases. Notably, and unexpectedly, the average sentiment of tweets in the Harris dataset is significantly more positive (*M* = .06) than those from the Pence dataset (*M* = -.02); (p < .001). In addition, we uncover online conversations that exhibit far-reaching network spread in which users express excitement and enthusiasm for the first-time candidacy of a woman of color as Vice-President. These upbeat messages likely account for a portion of the relatively positive average sentiment, and their sentiment score helps to offset the tone of the offensive tweets. For this job interview, we also know the outcome—Harris emerged as Vice-President on President Biden’s winning ticket—and affirming content should not be unexpected.

Findings for both Pence and Harris demonstrate the presence of gender and race stereotypes that emerge in the online assessments of each of the two candidates. Pence and Harris are not simply lauded or criticized for their political opinions, general knowledge, or their proposed national policies. Instead, commentary often focuses on their gender and race performance, employing demeaning stereotypes to assail their behaviors and character. Harris is subjected to substantially more of this type of targeting, but Pence’s masculinity and white heritage are not left unscathed. Pence is accused of being overly submissive (i.e., “for a man”), for example, and his whiteness is mocked. A common approach to denigrating these political candidates, thus, strikes at their gender and/or race presentation, aiming to render personal level hurt and embarrassment. The fact that harassers choose sexist and racist words to inflict damage underscores the salience of persistent race and gender typecasting in our society.

The bulk of our findings align with social theories of online aggression [47], which maintain that harassing messages reinforce traditional race and gender stereotypes and bolster the status of those who post negative content by attaining more retweets. Harassers routinely invoke well-worn, stereotypical insults in their attacks on the candidates, especially when targeting the woman of color. Furthermore, within the dataset for both candidates, the number of retweets (logged) relates significantly and negatively to sentiment, supporting the argument that cyberbullies gain attention online by posting particularly negative tweets. Note, too, that retweets for the minority, woman candidate are more negative, on average, and their spread was more extensive. These adverse messages range far in the virtual world.

Although there are several strengths to our study, there are also limitations. First, our dataset is limited to Twitter users, who are more urban, younger, and more diverse ethnically than the U.S. population [70]. Thus, our sample cannot be considered representative of the general population or their opinion. Although our sentiment classifier performed relatively well, moreover, we note that the meaning of tweets can be ambiguous and difficult to quantify. The use of gendered terms on Twitter can invoke multiple meanings, for example, such as regards the epithet “no homo” [71]. In addition, our final regression analyses suggest that unaccounted for dynamics likely contribute to tweet emotional content, given the unexplained variance in the models, and exploration of these dynamics is a topic for further inquiry. We also include only a subset of messages oriented towards the two candidates during the debate period, choosing to focus largely on those with hashtags that were, in theory, more neutral and/or comparable (e.g., #vpdebate). The addition of alternative, more idiosyncratic, hashtags likely would alter our conclusions (e.g., #flygate; #KamalaHarrisisafraid). Our estimates of the frequency of harassing race and gender material could be relatively conservative, for instance, given that the bulk of our dataset relied on neutral hashtags. Our results also call attention to the necessity for further scholarly work that just now is beginning to consider practices and policies to mitigate serious abuse on these platforms [27,28,32,72].

In conclusion, virtual reactions to the two candidates applying for the second highest leadership position within the U.S. government, that of Vice-President, highlight the ubiquitous and insidious nature of gender and race stereotypes, expressed through pejorative slurs. Both these demographic and cultural constructs successfully infiltrated commentary in this highly visible and noteworthy public debate. Given the heightened use of racist and masculine, sexist language in the tweets regarding Harris, the online interactions depict unequal standards applied to Harris and Pence. Findings highlight the underbelly of a social media platform, with its potential to be used as a tool to reify sexism and racism. Despite greater, overall positivity of tweets relating to Harris as compared to Pence, moreover, the presence of significantly more retweets about Harris, and more negativity of those retweets, demonstrate the potential for widespread dissemination of disparaging messages regarding the female, minority candidate.

Considering the relative permanence of content on Twitter [73], the pattern uncovered here remains disquieting. Unless removed by the individual or by the company, depreciatory messages can linger online indefinitely, continue to be retweeted, and promote the acceptance of public incivility, especially towards a minority, woman politician. Particularly troublesome is the possibility that online slurs will silence and deter promising women candidates, and those from underrepresented groups, from considering public office [32]. Given that Twitter affects news coverage [1], serves as an echo chamber for extremist opinions [56], and influences elections [3], a pressing concern is that pejorative messages ultimately will contribute to crucial, political outcomes. Future research needs to attend to the potential for individual and societal harm produced by this form of online harassment.

## Supporting information

S1 Table List of Tweet ID’s for all Tweets in the dataset. (RDS)

S2 Table Table containing the values of each variable for each tweet in the dataset. (RDS)

## References

[1] McGregor SC, Molyneux L. Twitter’s influence on news judgment: An experiment among journalists. Journalism. 2020 May;21(5):597–613. 10.1177/1464884918802975.

[2] Bond RM, Fariss CJ, Jones JJ, Kramer AD, Marlow C, Settle JE, et al. A 61-million-person experiment in social influence and political mobilization. Nature. 2012 Sep;489(7415):295–8. doi: 10.1038/nature11421 22972300 PMC3834737

[3] Fujiwara T, Müller K, Schwarz C. The effect of social media on elections: Evidence from the United States. National Bureau of Economic Research; 2021 May 31. 10.3386/w28849.

[4] Agudo U, Matute H. The influence of algorithms on political and dating decisions. PLoS One. 2021 Apr 21;16(4):e0249454. doi: 10.1371/journal.pone.0249454 33882073 PMC8059858

[5] Pager D, Shepherd H. The sociology of discrimination: Racial discrimination in employment, housing, credit, and consumer markets. Annu Rev Sociol. 2008 Aug 11;34:181–209. doi: 10.1146/annurev.soc.33.040406.131740 20689680 PMC2915460

[6] Gaddis SM. Discrimination in the credential society: An audit study of race and college selectivity in the labor market. Soc Forces. 2015 Jun 1;93(4):1451–79. 10.1093/sf/sou111.

[7] Zschirnt E, Ruedin D. Ethnic discrimination in hiring decisions: a meta-analysis of correspondence tests 1990–2015. J Ethn Migr Stud. 2016 May 27;42(7):1115–34. 10.1080/1369183X.2015.1133279.

[8] González MJ, Cortina C, Rodríguez J. The role of gender stereotypes in hiring: A field experiment. Euro Sociol Rev. 2019 Apr 1;35(2):187–204. 10.1093/esr/jcy055.

[9] Correll SJ, Benard S, Paik I. Getting a job: Is there a motherhood penalty?. AJS. 2007 Mar;112(5):1297–338. 10.1086/511799.

[10] Parker K, Funk C. Gender discrimination comes in many forms for today’s working women. Pew Research Center. 2017 Dec 14. Available from: https://www.pewresearch.org/fact-tank/2017/12/14/gender-discrimination-comes-in-many-forms-for-todays-working-women/.

[11] Brown A. The data on women leaders. Pew Research Center: Social. 2017. Available from: https://www.pewsocialtrends.org/fact-sheet/the-data-on-women-leaders/.

[12] Wojcik S, Hughes A. Sizing up Twitter users. Pew Research Center. 2019 Apr 24. Available from: https://www.pewresearch.org/internet/2019/04/24/sizing-up-twitter-users/.

[13] Lenhart A, Ybarra M, Zickuhr K, Price-Feeney M. Online harassment, digital abuse, and cyberstalking in America. Data & Society Research Institute. Center for Innovative Public Health Research. Available from: https://datasociety.net/pubs/oh/Online_Harassment_2016.pdf. 2016.

[14] Xu JM, Jun KS, Zhu X, Bellmore A. Learning from bullying traces in social media. Proceedings of the 2012 conference of the North American chapter of the association for computational linguistics: Human language technologies 2012 Jun. p. 656–66. Available from: http://dl.acm.org/citation.cfm?id=2382139.

[15] Felmlee D, Rodis PI, Francisco SC. What a B!tch!: Cyber aggression toward women of color. Gender and the media: Women’s places 2018 Nov 12. Emerald Publishing Limited. Advances in Gender Research 26:105–23. 10.1108/S1529-212620180000026008.

[16] Sterner G, Felmlee D. The social networks of cyberbullying on Twitter. Int J Technoethics (IJT). 2017 Jul 1;8(2):1–5. 10.4018/IJT.2017070101.

[17] Felmlee D, Rodis PI, Zhang A. Sexist slurs: Reinforcing feminine stereotypes online. Sex Roles. 2020 Jul;83(1):16–28. 10.1007/s11199-019-01095-z.

[18] Juvonen J, Gross EF. Extending the school grounds?—Bullying experiences in cyberspace. J Sch Health. 2008 Sep;78(9):496–505. doi: 10.1111/j.1746-1561.2008.00335.x 18786042

[19] Patchin JW, Hinduja S, editors. Cyberbullying prevention and response: Expert perspectives. Routledge; 2012 Mar 28. 10.1080/13632752.2012.706901.

[20] Orlando E, Saab A. Group Slurs, Stereotypes, and Speech Acts. Acta Analytica. 2020 Feb 13;35:599–621. 10.1007/s12136-020-00424-2.

[21] Vogels EA. The state of online harassment. Pew Research Center. 2021 Jan 13. Available from: https://www.pewresearch.org/internet/2021/01/13/the-state-of-online-harassment/.

[22] League AD. Online Hate and Harassment. The American Experience 2020. Center for Technology and Society. 2020. Available from: www.adl.org/media/14643/download.

[23] Marin M. Troll patrol findings. Amnesty International. 2018 Dec 18. Available from: https://decoders.amnesty.org/projects/troll-patrol/findings.

[24] Banet-Weiser S, Miltner KM. # MasculinitySoFragile: Culture, structure, and networked misogyny. Fem Media Stud. 2016 Jan 2;16(1):171–4. 10.1080/14680777.2016.1120490.

[25] Inara Rodis PD. Let’s (re) tweet about racism and sexism: responses to cyber aggression toward Black and Asian women. Inf Commun Soc. 2021 Oct 26;24(14):2153–73. 10.1080/1369118X.2021.1962948.

[26] Lewis R, Rowe M, Wiper C. Online abuse of feminists as an emerging form of violence against women and girls. Br J Criminol. 2017 Nov 1;57(6):1462–81. 10.1093/bjc/azw073.

[27] Felmlee D, DellaPosta D, Rodis PD, Matthews SA. Can Social Media Anti-abuse Policies Work? A Quasi-experimental Study of Online Sexist and Racist Slurs. Socius. 2020 6:1–16. 10.1177/2378023120948711.

[28] Jankowicz N, Hunchak J, Pavliuc A, Davies C, Pierson S, Kaufmann Z. Malign Creativity: How gender, sex and lies are weaponized against women online. Available from: https://www.wilsoncenter.org/publication/malign-creativity-how-gender-sex-and-lies-are-weaponized-against-women-online.

[29] Vickery JR. This isn’t new: gender, publics, and the Internet. Mediating Misogyny. Palgrave Macmillan:Cham; 2018. p. 31–49. 10.1007/978-3-319-72917-6_2.

[30] Baele SJ, Brace L, Coan TG. From “Incel” to “Saint”: Analyzing the violent worldview behind the 2018 Toronto attack. Terrorism and Political Violence. 2019 Aug 4:1–25. 10.1080/09546553.2019.1638256.

[31] Ging D. Alphas, betas, and incels: Theorizing the masculinities of the manosphere. Men Masc. 2019 Oct;22(4):638–57. 10.1177/1097184X17706401.

[32] Sobieraj S. Credible threat: Attacks against women online and the future of democracy. Oxford University Press; 2020 Sep 8.

[33] Guerin C, Maharasingam-Shah E. Public Figures, Public Rage: Candidate abuse on social media. ISD: Institute of Strategic Dialogue. 2020 Oct 5. Available from: https://www.isdglobal.org/wp-content/uploads/2020/10/Public-Figures-Public-Rage-4.pdf.

[34] Rheault L, Rayment E, Musulan A. Politicians in the line of fire: Incivility and the treatment of women on social media. Research & Politics. 2019 Jan;6(1):1–7. 10.1177/2053168018816228.

[35] Lawless JL. Female candidates and legislators. Annu Rev Polit Sci (Palo Alto). 2015 May 11;18:349–66. 10.1146/annurev-polisci-020614-094613.

[36] Dolan K. Gender stereotypes, candidate evaluations, and voting for women candidates: what really matters?. Polit Res Q. 2014 Mar;67(1):96–107. 10.1177/1065912913487949.

[37] Collins PH. Black feminist thought: Knowledge, consciousness, and the politics of empowerment. Routledge; 2002 Jun 1.

[38] Crenshaw K. Mapping the Margins: Intersectionality, Identity Politics, and Violence Against Women of Color. Stanford Law Rev. 1991 Jul;43(6):1241–99. 10.2307/1229039.

[39] Shields SA. Gender: An intersectionality perspective. Sex roles. 2008 Sep;59(5):301–11. 10.1007/s11199-008-9501-8.

[40] Bailey M. They aren’t talking about me. Crunk Feminist Collective. 2010 Mar 14;14. 10.1080/14680777.2016.1120490.

[41] Dowe PKF. The Community Matters: Finding the Source of the Radical Imagination of Black Women’s Political Ambition. J Women Polit Policy 2022 May 4;43(3):263–278. 10.1080/1554477X.2022.2070829.

[42] Dowe PKF. Resisting Marginalization: Black Women’s Political Ambition and Agency. PS Polit Sci Polit 2020 Oct;53(4):697–702. 10.1017/S1049096520000554.

[43] Carter NM, Dowe PKF. The Racial Exceptionalism of Barack Obama. J Afr Am Stud (New Brunsw) 2015;19(2):105–119. 10.1007/s12111-015-9298-9.

[44] Lemi DC. Do Voters Prefer Just Any Descriptive Representation? The Case of Multiracial Candidates. Perspectives on Politics 2021 Dec;19(4):1061–1081. 10.1017/S1537592720001280.

[45] Lemi DC, Arora M, Sadhwani S. Black and Desi: Indian American Perceptions of Kamala Harris. J Women Polit Policy 2022 May 4;43(3):376–389. 10.1080/1554477X.2022.2075678.

[46] Fuchs C. In Kamala Harris’ Presidential Campaign, Indian Americans Want More Opportunities to Connect.” NBC News. 2019 Feb 12. Available from: https://www.nbcnews.com/news/asian-america/kamala-harris-presidential-campaign-indian-americans-want-more-opportunities-connect-n965436.

[47] Felmlee D, Faris R. Toxic ties: Networks of friendship, dating, and cyber victimization. Soc Psychol Q. 2016 Sep;79(3):243–62. 10.1177/0190272516656585.

[48] Homans GC. The Human Group, New York: Harcourt, Brace & World.

[49] Horne C, Mollborn S. Norms: An integrated framework. Annu Rev Sociol. 2020 Jul 30;46:467–87. 10.1146/annurev-soc-121919-054658.

[50] Faris R, Felmlee D. Casualties of social combat: School networks of peer victimization and their consequences. Am Sociol Rev. 2014 Apr;79(2):228–57. 10.1177/0003122414524573.

[51] Salmivalli C. Bullying and the peer group: A review. Aggress Violent Behav. 2010 Mar 1;15(2):112–20. 10.1016/j.avb.2009.08.007.

[52] Gould RV. The origins of status hierarchies: A formal theory and empirical test. AJS. 2002 Mar;107(5):1143–78. 10.1086/341744.

[53] Zimmerman AG, Ybarra GJ. Online aggression: The influences of anonymity and social modeling. Psychol Pop Media Cult. 2016 Apr;5(2):181–93. 10.1037/ppm0000038.

[54] Olaniran B, Williams I. Social Media Effects: Hijacking Democracy and Civility. Civic Engagement. Platforms, Protests, and the Challenge of Networked Democracy Palgrave Macmillan:Cham; 2020. p. 77–94. 10.1007/978-3-030-36525-7_5.

[55] Moffitt B. Populism 2.0: Social media and the false allure of ‘unmediated’ representation. In: Moffit B. Populism and the Crisis of Democracy. Routledge; 2018. p. 30–46.

[56] Gaisbauer F, Pournaki A, Banisch S, Olbrich E. Ideological differences in engagement in public debate on Twitter. PLoS One. 2021 Mar 25;16(3):e0249241. doi: 10.1371/journal.pone.0249241 33765104 PMC7993819

[57] Flores RD. Do anti-immigrant laws shape public sentiment? A study of Arizona’s SB 1070 using Twitter data. AJS. 2017 Sep 1;123(2):333–84. 10.1086/692983.

[58] Salganik MJ. Bit by bit: Social research in the digital age. Princeton University Press;2019 Aug 6.

[59] Scarborough WJ. Feminist Twitter and Gender Attitudes: Opportunities and Limitations to Using Twitter in the Study of Public Opinion. Socius. 2018 Jun 5;4:1–16. 10.1177/2378023118780760.

[60] Omena JJ, Rabello ET, Mintz AG. Digital Methods for Hashtag Engagement Research. New Media Soc. Jul-Sep 2020;1–18 https://journals.sagepub.com/doi/pdf/10.1177/2056305120940697.

[61] Kearney MW. rtweet: Collecting Twitter data. R package version 0.6. 2018;7:1–72. Available from: https://cran.r-project.org/web/packages/rtweet/index.html.

[62] Wang W, Chen L, Thirunarayan K, Sheth AP. Cursing in english on twitter. Proceedings of the 17th ACM conference on Computer supported cooperative work & social computing 2014 Feb 15. p. 415–425. 10.1145/2531602.2531734.

[63] Yount KM, Sharma K. The US vice presidential debate: a Black woman’s resistance to white masculine dominance and white fragility to assert equal voice on public policy. J Gend Stud. 2021 Nov 17;30(8):964–70. 10.1080/09589236.2020.1867521.

[64] Zhang A, Felmlee D. You *&#*%!: Identifying bullying tweets. Poster presented at The 2017 Graduate Exhibition, University Park, PA.

[65] Hu M, Liu B. Mining and Summarizing Customer Reviews. KDD 2004. 10.1145/1014052.1014073.

[66] Nielsen FA. A new ANEW: Evaluation of a word list for sentiment analysis in microblogs. arXiv:1103.2903v1. 10.48550/arXiv.1103.2903.

[67] Felmlee D, Blanford J, Matthews S, MacEachren A. The geography of sentiment towards the Women’s Mach of 2017. PLoS One. 2020 June 4; 15(6): e0233994. 10.1371/journal.pone.0233994. 32497125 PMC7272063

[68] Freese J. Defending the Decimals: Why Foolishly False Precision Might Strengthen Social Science. Sociol Sci. 2014 Dec 1;1. 10.15195/v1.a29.

[69] Sayyadiharikandeh M, Varol O, Yang KC, Flammini A, Menczer F. Detection of novel social bots by ensembles of specialized classifiers. Proceedings of the 29th ACM International Conference on Information & Knowledge Management 2020 Oct 19. p. 2725–32. 10.1145/3340531.3412698.

[70] Smith A, Brenner J. Twitter use 2012. Pew Internet & American Life Project. 2012 May 31;4:1–2. Available from: https://www.pewinternet.org/2012/05/31/twitter-use-2012.

[71] Pascoe CJ, Diefendorf S. No homo: Gendered dimensions of homophobic epithets online. Sex Roles. 2019 Feb 1;80(3–4):123–36. 10.1007/s11199-018-0926-4.

[72] Barlett CP, DeWitt CC, Maronna B, Johnson K. Social media use as a tool to facilitate or reduce cyberbullying perpetration: A review focusing on anonymous and nonanonymous social media platforms. Violence Gend. 2018 Sep 1;5(3):147–52. 10.1089/vio.2017.0057.

[73] Julien C. Couch Revisited: A Theoretical Treatment of the Information‐Technological Media of Imgur, Reddit, and Twitter. Symb Interact. 2019 Feb;42(1):46–69. 10.1002/symb.403.

